# Supplementary material for: mLiftOver: harmonizing data across Infinium DNA methylation platforms
Source: Bioinformatics. 2024 Jul 4;40(7):btae423. doi: 10.1093/bioinformatics/btae423 (PMC11233119; doi:10.1093/bioinformatics/btae423)
Supplement: btae423_Supplementary_Data [file btae423_supplementary_data.pdf]

A

Tissue Type	HM450	EPIC
Blood	2000	2000
Bone	29	0
Bone marrow	281	72
Brain	2000	1967
Breast	288	0
Buccal cells	705	446
Esophagus	59	0
Fallopian tube	0	23
Fibroblast	333	327
Inner cell mass	55	0
Intestine	426	1246
Kidney	119	80
Liver	214	232
Lung	400	816
Muscle	88	518
Nasal	264	293
Placenta	1292	1514
Prostate	67	45
Saliva	509	305
Salivary gland	28	103
Skin	174	438
Sperm	339	178
Stomach	33	0
Umbilical cord	1023	28

B

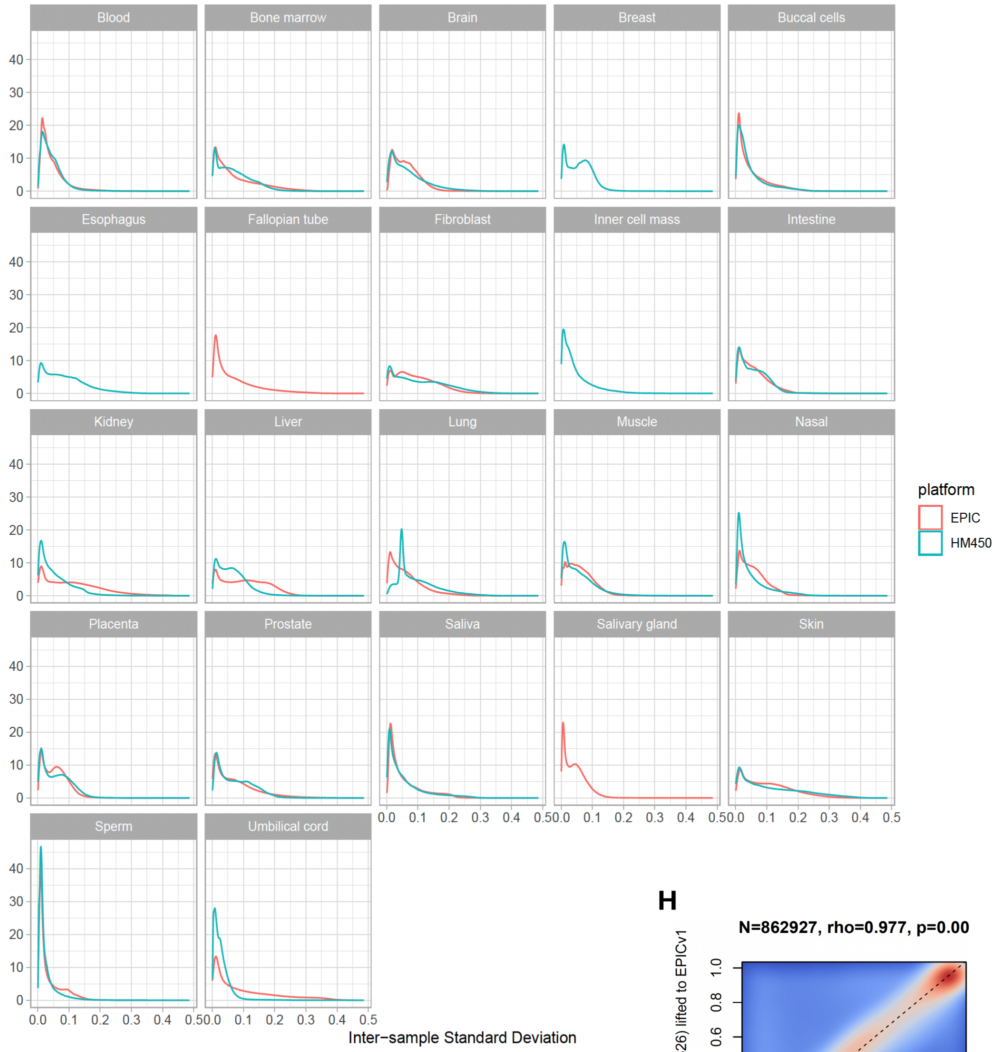

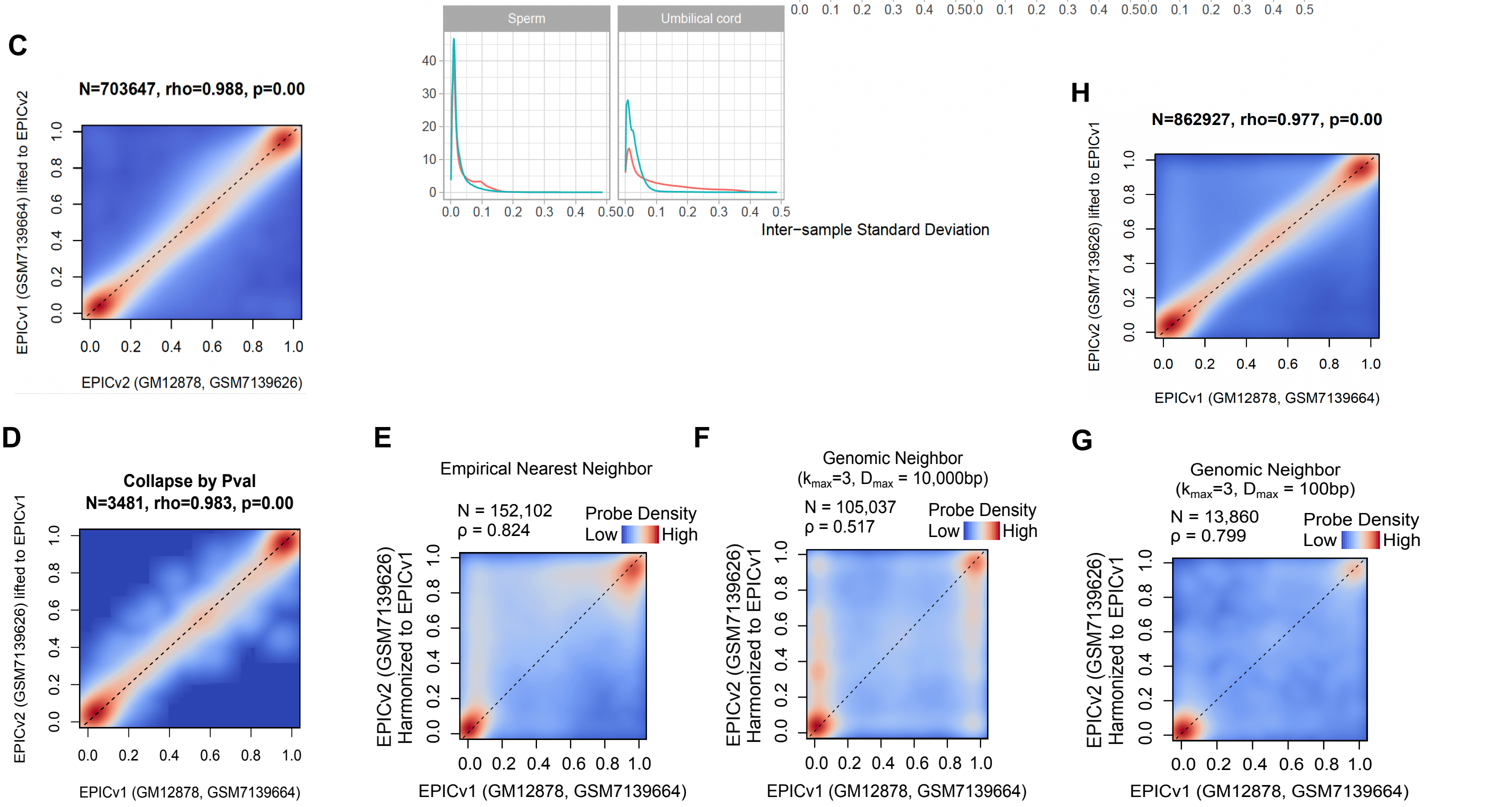

Supplemental Figure S1. mLiftOver harmonizes Infinium DNA methylation BeadChip data across array platforms. (A) Count of datasets (samples) used to derive baseline methylation imputation reference. (B) Methylation level standard deviation in datasets stratified by tissue type and Infinium platforms (HM450 and EPIC). (C) Evaluation of mLiftOver accuracy in translating cell line data from EPICv1 to EPICv2 (Y-axis), benchmarked against native EPICv2 data (X-axis). (D) Aggregation of replicate probes by minimal detection P-value. (E) imputation of missing probe readings without exclusion by methylation level standard deviation. (F,G) Comparison of empirical nearest neighbor imputation with genomic neighbor averaging with maximum distance 10kbp (F) and 100bp (G). (H) Overall accuracy, including both directly translated and imputed probe readings.
